# Human Brain Microvascular Endothelial Cells Derived from the BC1 iPS Cell Line Exhibit a Blood-Brain Barrier Phenotype

**DOI:** 10.1371/journal.pone.0152105

**Published:** 2016-04-12

**Authors:** Moriah E. Katt, Zinnia S. Xu, Sharon Gerecht, Peter C. Searson

**Affiliations:** 1 Department of Materials Science and Engineering, Johns Hopkins University, 3400 North Charles Street, Baltimore, MD, 21218, United States of America; 2 Institute of Nanobiotechnology, 100 Croft Hall, Johns Hopkins University, 3400 North Charles Street, Baltimore, MD, 21218, United States of America; 3 Department of Biomedical Engineering, Johns Hopkins University, 720 Rutland Avenue, Baltimore, MD, 21205, United States of America; 4 Department of Chemical and Biomolecular Engineering, Johns Hopkins University, 3400 North Charles Street, Baltimore, MD, 21218, United States of America; Hungarian Academy of Sciences, HUNGARY

## Abstract

The endothelial cells that form capillaries in the brain are highly specialized, with tight junctions that minimize paracellular transport and an array of broad-spectrum efflux pumps that make drug delivery to the brain extremely challenging. One of the major limitations in blood-brain barrier research and the development of drugs to treat central nervous system diseases is the lack of appropriate cell lines. Recent reports indicate that the derivation of human brain microvascular endothelial cells (hBMECs) from human induced pluripotent stem cells (iPSCs) may provide a solution to this problem. Here we demonstrate the derivation of hBMECs extended to two new human iPSC lines: BC1 and GFP-labeled BC1. These hBMECs highly express adherens and tight junction proteins VE-cadherin, ZO-1, occludin, and claudin-5. The addition of retinoic acid upregulates VE-cadherin expression, and results in a significant increase in transendothelial electrical resistance to physiological values. The permeabilities of tacrine, rhodamine 123, and Lucifer yellow are similar to values obtained for MDCK cells. The efflux ratio for rhodamine 123 across hBMECs is in the range 2–4 indicating polarization of efflux transporters. Using the rod assay to assess cell organization in small vessels and capillaries, we show that hBMECs resist elongation with decreasing diameter but show progressive axial alignment. The derivation of hBMECs with a blood-brain barrier phenotype from the BC1 cell line highlights that the protocol is robust. The expression of GFP in hBMECs derived from the BC1-GFP cell line provides an important new resource for BBB research.

## Introduction

The blood-brain barrier (BBB) is a dynamic and complex system responsible for maintaining homeostasis in the brain by regulating the chemical environment, immune cell transport, and the entry of toxins and pathogens [1, 2]. The microvascular endothelial cells that form the 600 km of capillaries in the human brain transduce biochemical and biomechanical signals between the vascular system and neurons, astrocytes, and pericytes in the brain [1, 2]. A major roadblock in blood-brain barrier research is the limited number of physiologically relevant cell types available for scientific discovery and translational studies [3–5]. Key characteristics of brain microvascular endothelial cells include: high transendothelial electrical resistance (TEER > 1000 Ω cm^2^), low permeability, and expression of tight junction proteins (e.g. claudin-5), transporters (e.g. LAT-1), and efflux pumps (e.g. P-gp) [6, 7].

Cells commonly used in BBB research include primary brain microvascular endothelial cells (BMECs) from vertebrate animals, type II Madin-Darby canine kidney cells (MDCK), immortalized human BMECs, and primary human brain microvascular endothelial cells (hBMECs) [8–10]. A fundamental problem in BBB research is that animal-derived cell lines and immortalized human BMECs do not fully recapitulate the characteristics of the human brain [6, 11, 12]. For example, the transendothelial electrical resistance of MDCK monolayers is typically around 200 Ω cm^2^, almost an order of magnitude lower than physiological values for brain microvasculature [6]. The disadvantages of primary hBMECs are that they are not readily available and lose some of their characteristics when cultured *in vitro* [13].

Stem cell derived hBMECs provide an alternative approach to producing cell lines for BBB research and drug discovery. Lippmann et al. have derived hBMECs from induced pluripotent stem cells (iPSCs), using the IMR90-4, DF6-9-9T, and DF19-9-11T cell lines, and from embryonic stem cells, using the H9 cell line [14]. IMR90-4 was induced from fetal fibroblasts using lentiviral vectors; DF6-9-9T and DF19-9-11T were both induced from foreskin fibroblasts using the oriP/EBNA-1 episomal vector [15, 16]. The robust differentiation takes just over a week and reproducibly produces hBMECs that express relevant tight junction proteins, transporters, and efflux pumps. Treatment of these derived cells with retinoic acid results in values of transendothelial electrical resistance in excess of 2000 Ω cm^2^ [17]. The derivation of brain-like endothelial cells from human hematopoietic stem cells has also been proposed as a source of cells for BBB research [18]. These cells exhibit many of the tight junction proteins and efflux pumps, but have low transendothelial electrical resistance and modest permeability.

The purpose of this study is to demonstrate that hBMECs can be derived from the BC1 human induced pluripotent stem cell line, using the approach developed by Lippmann et al. [14, 17]. The BC1 cell line uses human feeder cells to avoid viral contamination and undesired immunogenicity, and achieves efficient reprogramming with a single transfection using the oriP/EBNA-1 plasmid [19, 20]. The reprogramming of the BC1 and BC1-GFP lines can be performed under xeno-free conditions, allowing translation for clinical applications [20]. The hBMECs derived from the BC1 cell line show a very similar phenotype to that reported by Lippmann et al., showing that the protocol is robust and generally applicable for BBB research. We also show the derivation of fluorescently-labeled hBMECs from the BC1-GFP cell line, providing an important new resource for individual cell tracking using live cell microscopy and identification of hBMECs in co-culture models. Finally, using a rod assay we show that the BC1-derived hBMECs will wrap around and form tight junctions with themselves and their upstream and downstream neighbors at diameters corresponding to capillary dimensions.

## Materials and Methods

### Cell Culture

BC1 [19] and BC1-GFP [21] human iPSCs (provided by Dr. Linzhao Cheng, Johns Hopkins University) were cultured on 40 μg mL^-1^ Matrigel-treated tissue culture dishes (Corning) in TeSR-E8 media (Stem Cell Technologies) from passage 45–70. The iPSCs were maintained in TeSR-E8 media which was changed daily, and passaged using StemPro® Accutase® solution (Life Technologies). 10 μM ROCK inhibitor (ATCC) was added to the culture media for 24 hours after passaging. The differentiation followed the protocol reported by Lippmann et al. [14, 17]. Briefly, iPSCs are cultured for 3–4 days then switched to unconditioned media without bFGF (UM/F-). The unconditioned media was DMEM/F12 (Life Technologies) supplemented with 20% KOSR (Life Technologies), 1% non-essential amino acids (Life Technologies), 0.5% L-glutamine (Sigma), and 0.836 μM beta-mercaptoethanol (Life Technologies). The cells were cultured in unconditioned media for 6 (BC1 cells) or 7 (BC1-GFP cells) days. Subsequently, the cells were switched to endothelial cell serum-free media (EC) (Life Technologies) supplemented with 1% human platelet poor derived serum (Sigma) and 20 ng mL^-1^ bFGF (R&D Systems). This EC media was also supplemented with 10 μM all-trans retinoic acid (Sigma) where indicated. Cells were cultured in T25 and T75 flasks (BD Falcon) with 4 or 12 mL of media respectively. Media was changed every 24 hours throughout the differentiation and all subsequent experiments.

Cells remained in EC media with or without retinoic acid for 2 days before being sub-cultured on either tissue culture dishes, 24 well (6.5 mm) Corning transwell polyester membranes with 0.4 μm pores, or glass rods, coated with a 50/50 mixture of 100 μg mL^-1^ collagen IV (Sigma) and 50 μg mL^-1^ fibronectin (Sigma) overnight. The same EC media with or without retinoic acid was used for all subsequent experiments. Cells were sub-cultured at a density of 1 x 10^6^ cells mL^-1^ for all subsequent experiments.

### Immunocytochemistry

Cells were washed once with DPBS and fixed with 3.7% paraformaldehyde for 5–10 minutes. The cells were then washed three times with DPBS and permeabilized with 0.1% Triton X-100 for 5–10 minutes. Following membrane permeabilization, cells were washed 3 times with DPBS and blocked with 10% donkey serum in DPBS (EMD Millipore) at room temperature for one hour. Cells were incubated with primary antibodies (**S1 Table**) for one hour at room temperature or overnight at 4˚C. After incubation with primary antibodies, the cells were washed three times with DPBS and incubated with secondary antibodies (Life Technologies) for one hour at room temperature. Epifluorescence images were taken with a Nikon TE-2000 Microscope using NIS Advanced Research software.

### Flow Cytometry

Two days after sub-culturing into tissue culture flasks, confluent monolayers of cells were washed once with DPBS and dissociated with StemPro® Accutase® for five minutes. Cells were fixed in 3.7% paraformaldehyde for 10 minutes and blocked with DPBS with 0.1% bovine serum albumin (BSA) overnight at 4˚C. Cells were incubated with primary antibodies (**S1 Table**) in DPBS with 0.1% BSA for one hour. IgG isotype controls were used at a matching concentration. After cells were washed three times with DPBS with 0.1% BSA, cells were incubated with donkey anti-rabbit Alexa Fluor® 488 (ThermoFisher) or donkey anti-mouse Alexa Fluor® 488 (ThermoFisher) or donkey anti-goat Alexa Fluor® 488 (ThermoFisher) in DPBS with 10% donkey serum and 0.1% BSA at 1:100 dilution for one hour at room temperature. After three washes with DPBS with 0.1% BSA, cells were analyzed on a BD FACSCalibur flow cytometer and the IgG and secondary controls were used to quantify positive labeling. GLUT-1^+^ cells were quantified based on GLUT-1 expression measured from GLUT-1-expressing HT-1080 cells and from flow cytometry compensation beads (ThermoFisher).

### Western Blot

Confluent monolayers of cells were lysed two days after sub-culturing in tissue culture flasks using RIPA buffer (Sigma) containing protease inhibitor cocktail (Sigma), centrifuged at 25000 RPM for 25 minutes at 4˚C, and stored at -20˚C. The protein concentration was quantified with an RC DC protein assay kit (Bio-Rad) and samples were normalized to the lowest concentration. Western blots were run on 4–15% pre-cast polyacrylamide gels (Bio-Rad), transferred to nitrocellulose membranes (Bio-Rad), and blocked with 5% fat-free skim milk (Bio-Rad) in TBST (Corning) with 0.05% TWEEN-20 (Sigma) for one hour at room temperature. Primary antibodies (**S1 Table)** were added to the milk cocktail and incubated for one hour at room temperature and overnight at 4˚C. Membranes were washed three times for 5 minutes each with TBS with 0.05% TWEEN-20. Secondary HRP antibodies (Bio-Rad) were added to milk and incubated for one hour at room temperature before imaging (Bio-Rad molecular imager ChemiDoc XRS+) using ImageLab 5.1 software. β-actin was used as a loading control. Experiments were run with duplicate samples on each gel. Western blots were performed in triplicate using lysate from three independent differentiations. An independent differentiation is defined as the complete differentiation from iPSCs to hBMECs beginning with a different passage of BC1 or BC1 GFP cells. Analysis of the relative intensities of the bands was performed using Image J. Each lane of the gel was normalized and compared to the intensity of the first hBMEC lane for analysis, with the exception of VE-cadherin, which was normalized to the intensity of the first hBMEC RA lane to reduce the effect of the background on the gel. The band selected for analysis on P-glycoprotein (P-gp) gels was around 150 kDa, as P-gp is a 150–170 kDa protein depending on phosphorylation level [22]. Although a significant amount of non-specific binding was observed, the 150 kDa band was consistent across all differentiations [22]. Full western blots are shown in **S1 Fig**. Western blots were performed for SOX-2 before and after the differentiation on BC1 and hBMEC RA cells to ensure the loss of stem cell markers (**S1 Fig**).

### Real-time Quantitative PCR (qPCR)

Quantitative PCR (qPCR) was performed to assess changes in gene expression in the following genes: *ABCB1*, *ABCC1*, *ABCG2*, *CDH5*, *CLDN5*, *OCLN*, *SLC2A1*, *SLC7A5*, *TJP1* with *ACTB* as the housekeeping gene. Cells were washed twice in cold DPBS, dissociated with StemPro® Accutase®, and lysed with the Cells-to-CT lysing solution (Life Technologies). PCR samples were prepared using the TaqMan® Gene Expression Cells-to-CT^TM^ Kit (Life Technologies). qPCR was performed using TaqMan® probes on an Applied Biosystems StepOnePlus Real-Time PCR system. Fold changes were obtained using the comparative CT method (ΔΔC_t_) normalizing to *ACTB* expression and comparing to the BC1-derived hBMECs (no retinoic acid) as the reference, and checked manually for claudin-5 using the algorithm in the Applied Biosystems User Bulletin: Relative Quantitation (RQ) algorithms in the Applied Biosystems Real-Time PCR Systems Software (July 2007).

### TEER and Permeability

Transendothelial electrical resistance and permeability measurements were used to characterize confluent monolayers. Cells were sub-cultured on 24 well Corning transwell polyester membranes. TEER measurements were recorded daily (EndOhm-6) beginning 24 hours after subculture on membranes. TEER measurements are corrected to a control well containing media alone and normalized by the membrane area.

Permeability across confluent monolayers was measured two days after sub-culture on membranes for Lucifer yellow (LY) (Life Technologies), tacrine (9-Amino-1,2,3,4-tetrahydroacridine hydrochloride hydrate; Sigma), and rhodamine 123 (Invitrogen). 100 μM LY, 3 μM tacrine, or 10 μM rhodamine 123 in 100 μL of transport buffer (distilled water with 0.12 M NaCl, 25 mM NaHCO_3_, 3 mM KCl, 2 mM MgSO_4_, 2 mM CaCl_2_, 0.4 mM K_2_HPO_4_, 1 mM HEPES, and 0.1% human platelet poor derived serum) was added to the apical compartment of the transwell dish and placed in the incubator. The basolateral well contained 600 μL of transport buffer. Every fifteen minutes the plate was removed from the incubator and the top well moved to a different bottom well containing transport buffer and placed back into the incubator. After one hour the buffer in the bottom well was collected and stored at 4˚C. For basolateral-to-apical measurements, 10 μM rhodamine 123 or 100 μM LY in 600 μL of transport buffer was added to the basolateral compartment, and 100 μL of transport buffer was added to the apical compartment. At 30 and 60 minutes, the buffer in the apical compartment was collected and stored at 4˚C. Samples were then measured on a fluorometer. Calibration curves were obtained for each experiment over a concentration range spanning four orders of magnitude starting at the initial concentration. The apparent permeability was calculated using previously reported methods [8]. Efflux ratios were calculated from the ratio of basolateral-to-apical and apical-to-basolateral permeabilities.

### Rod Assay

To assess the response of the BC1-derived hBMECs to the high curvature typical of brain capillaries, we used the rod assay [23]. Glass rods (0.125 inches in diameter) were pulled to various diameters between 10 μm and 200 μm under a hot flame and separated into 2 cm sections. Rods with visible defects observed under an optical microscope were discarded. Glass rods of similar diameter were mounted onto double-sided tape supports on a 2 cm x 2 cm glass cover slip at 1.5 mm apart. The assembly was placed into a petri dish and sterilized in ethanol for 20 minutes. The rods were then coated with a 50/50 mixture of 100 μg mL^-1^ collagen IV (BD Biosciences) and 50 μg mL^-1^ fibronectin (BD Biosciences). Cells were seeded on the rods at approximately 1 x 10^6^ cells mL^-1^ and allowed to grow to confluence, from a few days to a week depending on the rod diameter. The cells were cultured in endothelial cell media with and without retinoic acid. Media was changed daily.

After reaching confluence, the cells on the rods were fixed and stained for DAPI and ZO-1 (Life Technologies). The rods were carefully removed from the glass slide set-up, placed in a 170 μm thick glass bottom petri dish and immersed in 2, 2′-thiodiethanol (Sigma Aldrich) to reduce the refractive index mismatch during microscopy and hence minimize distortion of the images [23, 24]. Z-stacks of the rods were taken in 0.8 μm steps using a confocal microscope with a 40x oil-immersion objective.

To analyze cell morphology, the 3D projection of the cells on rods were transformed to a 2D plane using UNWRAP, a program that takes the surface of the cylindrical rod and unwraps the image onto a 2D plane [23]. Cell-cell junctions were traced using ImageJ to collect cell morphology details, including the inverse aspect ratio (a measure of elongation defined by the length of short axis divided by the length of the long axis), the average orientation angle (a measure of alignment defined by the angle between the long axis of the cell and the rod axis), and the cell area (μm^2^) (**S3 Fig**). The number of cells wrapping around the perimeter of the rods (N_cell_) was calculated by drawing a line perpendicular to the rod axis in the unwrapped images and counting number of cells crossing the line. The lines were drawn far enough apart so that no cells were counted more than once.

## Results

### Differentiation of BC1 and BC1-GFP iPSCs into human brain microvascular endothelial cells (hBMECs)

**Fig 1:**
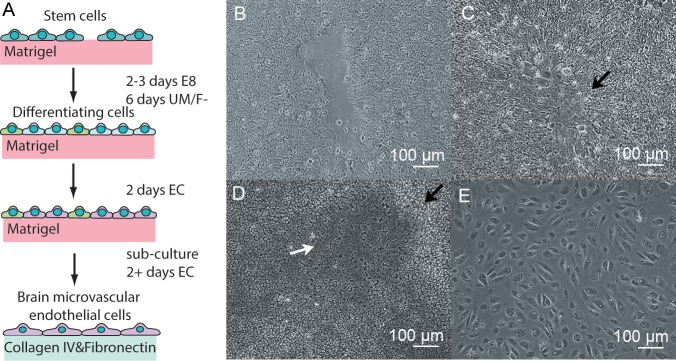
Differentiation of BC1 iPSCs without retinoic acid. (A) Schematic illustration of the stages of differentiation. (B) The expansion and flattening of colonies at the beginning of step 1. (C) The formation of neural rosettes at the junction between colonies around day 3 in step 1. (D) Clearly visible regions of endothelial cells with a cobblestone morphology, indicated by the white arrow, separated by neural tracts, indicated by the black arrow, at the end of step 1. (E) Endothelial cells 24 hours after subculture in step 3. Brightness and contrast were adjusted for clarity in (B-E).

BC1 and BC1-GFP iPSCs were differentiated into hBMECs using a slightly modified version of the three-step procedure developed by Lippmann et al. [14, 17] (**Fig 1A**): (1) differentiation of iPSCs in UM/F- media, (2) selective maturation of the endothelial cells in EC media, and (3) purification of the endothelial cells by sub-culturing on collagen IV and fibronectin coated surfaces. For the BC1-GFP cell line, the length of step 1 was increased by one day. The progression of the differentiation process is shown in **Fig 1B–1E**. The iPSCs are cultured until reaching about 70% confluence. After changing to UM/F- media (step 1), the iPSC colonies flatten and grow together (**Fig 1B**). Neural rosettes form at the boundaries between colonies, and neural progenitor cells begin to develop and form a network of neural tracts throughout the plate (**Fig 1C**). In between these neural tracts, endothelial-like cells appear with a characteristic cobblestone morphology (**Fig 1D)**. When the endothelial-like cells are clearly visible, after 6 days for the BC1 cells and 7 days for the BC1-GFP cells, the media is changed to EC media with or without retinoic acid to promote further maturation and development of the endothelial-like cells at the expense of the neural tracts (step 2). After two days in EC media, the cells are then sub-cultured on collagen IV and fibronectin coated surfaces (step 3). Since the endothelial cells are more adhesive than other cells to the collagen IV and fibronectin coated surface, non-adhesive cells are removed when the media is changed (every 24 h), thereby increasing the purity. To assess the influence of retinoic acid on the derived cells, 10 μM RA was added to the EC media for selective maturation (step 2), and sub-culture (step 3).

The differentiation is highly reproducible, indicating that the procedure developed by Lippmann et al. is robust and applicable to other iPS cell lines. During the differentiation we found two steps to be critical for successful differentiation. (1) The time at which step 1 is initiated (i.e. switching to UM/F- media) following seeding of the iPSCs onto the Matrigel-coated plates is critical. The differentiation should be started when the iPSCs reach approximately 70% confluence. If the differentiation is started too early the colonies will take longer to grow together and in the process fibroblast-like cells will begin to develop, which are difficult to remove during the sub-culture step. Conversely, if the differentiation is started too late the colonies will grow together too rapidly and the resulting neural tracts will be fewer and the resulting ECs will have a less brain-like phenotype [25]. (2) The length of step 1 is also critical. The transition to step 2 (i.e. switch to EC media) should occur when endothelial-like cells are clearly visible. If the transition to step 2 occurs too early, there will not be a sufficient density of endothelial-like cells to develop into mature endothelial cells, resulting in a low yield from the differentiation.

### Characterization: tight junction expression and localization

A characteristic feature of brain microvascular endothelial cells is the formation of tight junctions that effectively eliminate paracellular transport across the blood-brain barrier [1, 26–28]. The expression of ZO-1, occludin, and claudin-5 are commonly used as markers of tight junction formation in monolayers of brain microvascular endothelial cells [26]. ZO-1 is a junctional adhesion protein that serves as a scaffold protein to anchor occludins and claudins to the actin cytoskeleton in the cell [29]. Occludin is a 60 kDa tight junction protein that directly binds to ZO-1 and contributes to the barrier functions of the tight junctions [29]. The claudin family consists of more than 20 proteins that are essential for tight junction formation; claudin-5 is the isoform most commonly found in the BBB [30–32]. Homotypic interactions between the two extracellular loops of claudin-5 are thought to play a key role in the formation of tight junctions [33, 34]. Immunocytochemistry was used to evaluate the expression and localization of tight junction proteins, and to assess whether GFP expression was maintained through the differentiation of BC1-GFP cells. Cells were seeded onto collagen IV and fibronectin coated glass bottom dishes and cultured for 2 days (step 3) to reach confluence before fixing and staining.

**Fig 2:**
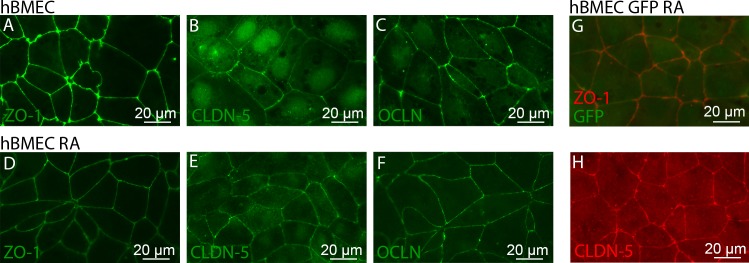
BC1-derived hBMECs show co-localization of ZO-1, CLDN-5, and OCLN at cell-cell junctions in confluent monolayers. hBMECs derived from the BC1-GFP cell line retain GFP expression throughout differentiation. (A-F) Junctional staining of hBMECs derived from the BC1 cell line, (A-C) without and (D-F) with the addition of retinoic acid. (G,H) Junctional staining of hBMECs derived from the BC1-GFP cell line. (A,D) ZO-1 (green), (B,E) CLDN-5 (green), and (C,F) OCLN (green). (G) ZO-1 (red) and GFP expression (green) (H) CLDN-5 (red). The look-up tables have been modified for improved visualization of the junctional stains for CLDN-5 (B,E) and OCLN (C,F).

We found that the tight junction proteins ZO-1, occludin, and claudin-5 all localize to the cell-cell junctions (**Fig 2**). Overlays of the images (not shown) confirm that these proteins are co-localized at the cell-cell junctions. ZO-1 is strongly localized at the cell junctions in hBMECs with and without retinoic acid (**Fig 2A and 2D**). Claudin-5, however, displays localization at the cell junctions and expression in the nucleus (**Fig 2B and 2E**). Some claudins have been reported to show nuclear localization [35]. Occludin is primarily expressed at the junctions, but less intensely than ZO-1, with a low level of intracellular expression (**Fig 2C and 2F**). The expression profiles of the tight junction proteins between hBMECs with and without retinoic acid are indistinguishable.

In hBMECs, both with and without retinoic acid, the cell-cell junctions appear relatively straight resulting in cells that appear polygonal rather than rounded or elliptical. The hBMECs derived without retinoic acid treatment (**Fig 2A–2C**) have a small population of cell-cell junctions that appear frayed, similar to observations by Lippmann et al. [17]. hBMECs treated with retinoic acid (**Fig 2D–2F**) have a smaller fraction of these defects.

The hBMECs derived from BC1-GFP cells maintain their GFP expression throughout the differentiation as well as expressing junctional proteins, including ZO-1 (**Fig 2G**). ZO-1 and claudin-5 expression in hBMECs derived from BC1 and BC1-GFP cells are indistinguishable (**Fig 2G and 2H)**. Similar to hBMECs derived from the BC1 cell line, hBMECs derived from BC1-GFP cells with the addition of retinoic acid had fewer defects in the cell-cell junctions.

### Characterization: protein expression

**Fig 3:**
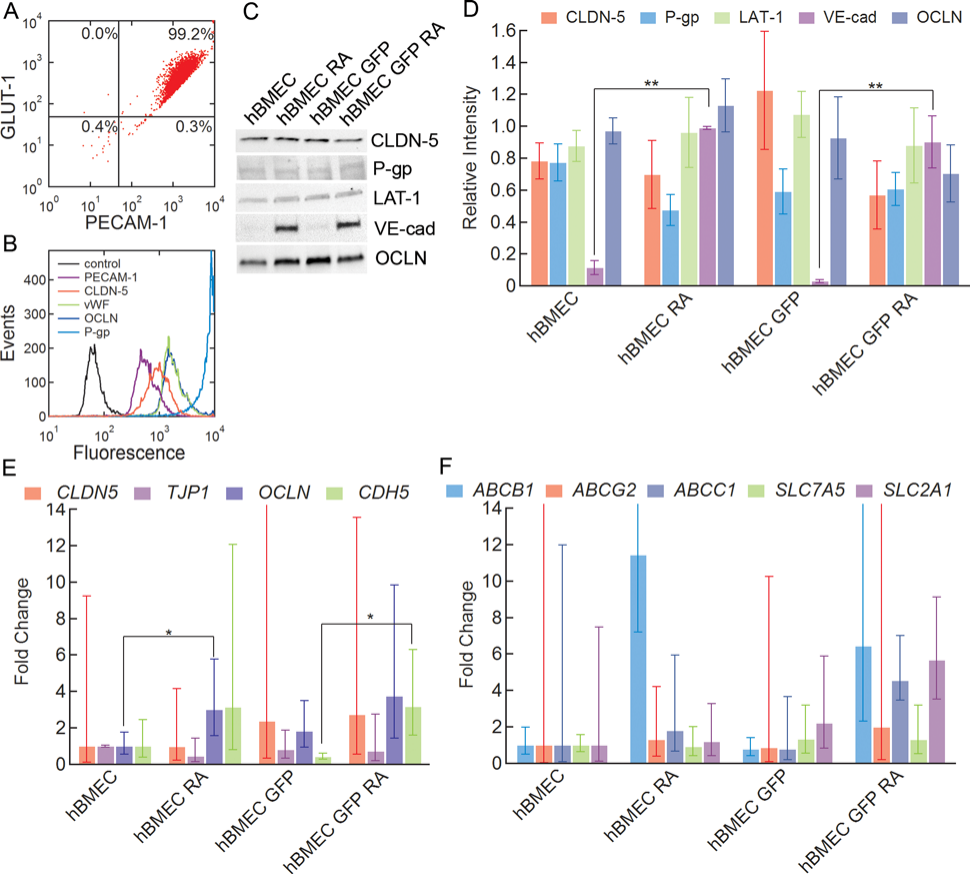
Protein and gene expression for confluent monolayers of hBMECs derived from the BC1 and BC1-GFP cell lines. (A) Representative flow cytometry dot plot of GLUT-1 and PECAM-1 populations in RA-treated BC1-derived hBMEC population. Mean ± SE. N = 3. (B) Representative flow cytometry histograms of PECAM-1, CLDN-5, vWF, OCLN, P-gp. (C) Representative western blot for CLDN-5, P-gp, LAT-1, VE-cad, and OCLN for hBMECs derived from BC1 cells (hBMEC), hBMECs derived from BC1 cells with retinoic acid (hBMEC RA), hBMECs derived from BC1-GFP cells (hBMEC GFP), and hBMECs derived from BC1-GFP cells with retinoic acid (hBMEC GFP RA). (D) Relative intensities compared to the intensity of the first hBMEC lane (see *Methods* for further details) of CLDN-5, P-gp, LAT-1, and OCLN, and relative intensity compared to the first hBMEC RA lane of VE-cad for hBMEC, hBMEC RA, hBMEC GFP, hBMEC GFP RA. Data were obtained from analysis of three separate differentiations. Error bars represent SE. (E) qPCR amplification plot for expression of junctional proteins: *CDH5*, *CLDN5*, *TJP1*, and *OCLN*. Data were obtained from analysis of three separate differentiations. Fold changes are with respect to the hBMEC cell line. Error bars represent SE. * represents p < 0.05, ** represents p < 0.01. (F) qPCR amplification plot for expression of efflux pumps and transporters: *ABCB1*, *ABCC1*, *ABCG2*, *SLC2A1*, and *SLC7A5*. Data were obtained from analysis of three separate differentiations. Fold changes are with respect to the hBMEC cell line. Error bars represent SE. * represents p < 0.05, ** represents p < 0.01.

The expression of important BBB proteins was assessed using flow cytometry and western blot. Flow cytometry dot plots of PECAM-1 and GLUT-1 populations in RA-treated hBMECs show 98.3 ± 1.2% PECAM-1^+^/GLUT-1^+^ cells, indicating a very high purity of PECAM-1^+^ endothelial cells with elevated GLUT-1^+^ expression (**Fig 3A**). Flow cytometry was also performed on endothelial, junctional, and transporter proteins: platelet endothelial cell adhesion molecule (PECAM-1), claudin-5 (CLDN-5), von Willebrand factor (vWF), occludin (OCLN), and P-glycoprotein (P-gp) (**Fig 3B**). Flow cytometry histograms show expression of these key endothelial and BMEC-phenotype markers in the derived hBMECs.

Western blots were performed to quantify the expression of claudin-5 (CLDN-5), P-glycoprotein (P-gp), large amino acid transporter 1 (LAT-1), vascular endothelial cadherin (VE-cad), and occludin (OCLN). P-glycoprotein (P-gp) is an ATP-dependent efflux pump highly expressed in BBB endothelial cells that is responsible for pumping out a broad range of small molecules and is one of the main reasons for the poor penetration of many small molecule drugs into the brain [7, 18]. The large amino acid transporter 1 (LAT-1) is the main transporter for large neutral amino acids and is highly expressed in the BBB [7, 36]. Vascular endothelial cadherin (VE-cad) is an adherens junction protein specific to endothelial cells [37].

The expression of all five proteins was clearly seen in hBMECs derived from both stem cell lines with and without the addition of retinoic acid (**Fig 3C** and **S1 Fig**). Quantitative analyses of expression levels for CLDN-5, P-gp, LAT-1, and OCLN show no statistical difference between cells derived from the BC1 and BC1-GFP cell lines, or with or without the addition of retinoic acid (p > 0.05) (**Fig 3D**). The expression of CLDN-5 and OCLN was reported for iPSC-derived hBMECs by Lippmann et al. [17]. CLDN-5 expression is a hallmark of hBMECs, but expressed at very low levels or not detected at all in immortalized hBMECs [11]. In contrast to CLDN-5, P-gp, LAT-1, and OCLN, the expression of VE-cad was significantly upregulated with the addition of retinoic acid (p < 0.01) for both BC1 and BC1-GFP cells (**Fig 3D**). The upregulation of VE-cad with retinoic acid treatment was also reported by Lippmann et al. [17].

### Characterization: gene expression

To further determine whether there is a difference among differentiations, the relative gene expressions of hBMEC junctional proteins, pumps, and transporters were analyzed. Quantitative PCR was performed on hBMECs to determine mRNA expression of nine genes, including four junctional protein genes: *CLDN5* (CLDN-5), *TJP1* (ZO1), *OCLN* (OCLN), and *CDH5* (VE-cad), and five transporter genes: *ABCB1* (P-gp), *ABCG2* (BCRP), *ABCC1* (MRP1), *SLC2A1* (GLUT-1), and *SLC7A5* (LAT-1), for both BC1 and BC1-GFP cells with and without retinoic acid treatment (**Fig 3E and 3F**). All fold changes are reported with respect to BC1-derived hBMECs without retinoic acid. With respect to junctional proteins, *OCLN* shows a significant difference in gene expression with and without retinoic acid for hBMECs derived from the BC1 cell line only (p < 0.05), while *CDH5* (VE-cad) shows a significant difference in gene expression with the addition of retinoic for the GFP-tagged (BC1-GFP) cell line (p < 0.05). There is no significant difference in expression of *CLDN5* and *TJP1* between hBMECs derived from BC1 and BC1-GFP cells, or between cells with or without retinoic acid. When examining the gene expression of efflux pumps and transporters, there is no significant difference due to the addition of retinoic acid in hBMECs derived from either cell line (p ≥ 0.05). The mean fold difference in *ABCB1* (P-gp) is several fold higher with the addition of retinoic acid in both the BC1-derived and BC1-GFP-derived hBMEC cell lines, although this increase is not statistically significant due to the high variability (p = 0.07). This trend is also seen in other efflux pumps, such as *ABCG2* and *ABCC1*, although to a lesser extent. Similar studies using IMR90-4 iPSC-derived hBMECs also show elevated *ABCB1*, *ABCG2*, and *ABCC1* expression with retinoic acid treatment [17]. The average *CLDN5* expression appears to be slightly higher in BC1-GFP-derived hBMECs with and without retinoic acid as compared to BC1-derived hBMECs, however, this is not statistically significant due to the very high variability between differentiations. The expression of *SLC7A5* (LAT-1) and *CLDN5* (CLDN-5) both increased for hematopoietic stem cell derived brain-like ECs when co-cultured with pericytes [18]. The large standard error for *ABCB1*, *ABCG2*, *ABCC1*, *SLC2A1*, and *CLDN5* could be due to differences in mRNA expression between differentiations.

### Functionality: TEER and permeability

**Fig 4:**
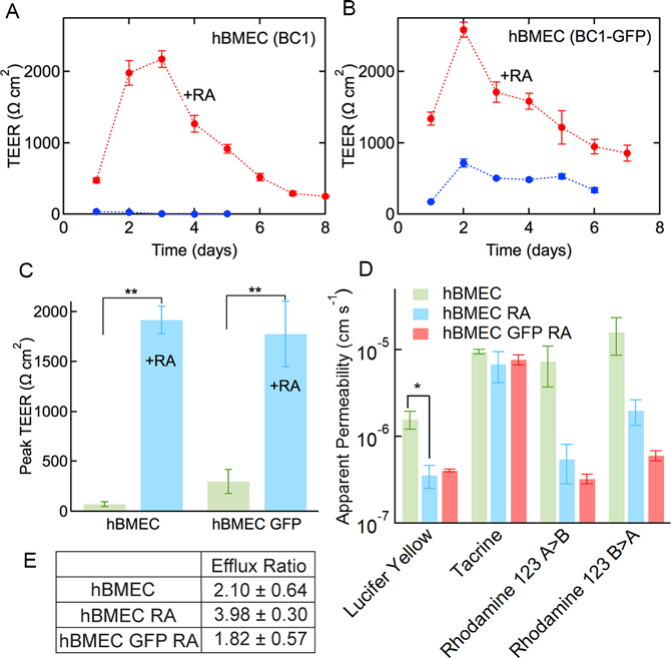
Transendothelial electrical resistance (TEER) and permeability of hBMECs with and without retinoic acid treatment. (A-B) Representative TEER plots for hBMEC, hBMEC RA, hBMEC GFP, and hBMEC GFP RA cells. Data were obtained from 5 independent measurements from one differentiation. Error bars represent SE. (C) Peak TEER values. Data were obtained from five separate differentiations. Error bars represent SE. (D) Permeability values for LY (data from four independent differentiations for BC1-derived cells and three independent differentiations for BC1 GFP-derived cells), tacrine (data from three independent differentiations), rhodamine 123 apical-to-basolateral (data from three independent differentiations), and rhodamine 123 basolateral-to-apical. (E) Efflux ratio of rhodamine 123 for hBMEC, hBMEC RA, and hBMEC GFP RA. Error bars represent SE. * represents p < 0.05, ** represents p < 0.01.

High transendothelial electrical resistance (TEER) is a hallmark of brain capillaries and is lacking from most cells used for BBB research [11, 38]. Measurements were recorded for hBMECs derived from BC1 and BC1-GFP cells with and without retinoic acid as a function of time after sub-culture (step 3) (**Fig 4A and 4B**). Peak TEER values for hBMECs derived from the BC1 and BC1-GFP cells without retinoic acid were 73.2 ± 22.9 Ω cm^2^ and 300 ± 120 Ω cm^2^, respectively (**Fig 4C**). With the addition of retinoic acid, these values increased to 1920 ± 139 Ω cm^2^ and 1780 ± 325 Ω cm^2^, respectively (p < 0.01). However, there was no statistical difference between hBMECs derived from BC1 and BC1-GFP cell lines (p > 0.05) with or without retinoic acid.

The addition of retinoic acid results in a statistically significant increase in peak TEER to values comparable to those reported for rat brains *in vivo* (1000–1500 Ω cm^2^) [38]. The TEER values are somewhat smaller than values of 2940 ± 800 Ω cm^2^ reported by Lippmann et al. for iPSC-derived hBMECs treated with retinoic acid, although these measurements were obtained in 12 well inserts [17]. In contrast, TEER values for MDCK monolayers are about 200 Ω cm^2^ and values for immortalized cell lines are even lower [2, 6]. TEER values of around 200 Ω cm^2^ have also been reported for hBMECs derived from hematopoietic stem cells co-cultured with pericytes [18].

The permeability of hBMECs derived from the BC1 cell line with and without retinoic acid, as well as the BC1-GFP-derived hBMECs cultured with retinoic acid, was assessed for 100 μM Lucifer yellow (LY), 3 μM tacrine (9-Amino-1,2,3,4-tetrahydroacridine hydrochloride hydrate), and 10 μM rhodamine 123 in a standard transwell assay. LY is a small negatively charged molecule (MW 444.3 g mol^-1^) that is often used to confirm the integrity of MDCK and BMEC monolayers prior to measuring solute permeability [39]. Tacrine (MW 198.3 g mol^-1^) is a cholinesterase inhibitor that has been used for the treatment of Alzheimer’s disease [40] and is convenient for permeability measurements due to its intrinsic fluorescence [41]. Rhodamine 123 (MW 380.8 g mol^-1^) is a well-known cationic efflux transporter substrate, and is commonly used to probe the functional activity of the P-gp pump [42, 43].

The permeability of LY was 1.58 ± 0.37 x 10^−6^ cm s^-1^ for hBMECs, 3.71 ± 1.1 x 10^−7^ cm s^-1^ for hBMECs with the addition of retinoic acid (N = 4), and 4.00 ± 0.15 x 10^−7^ cm s^-1^ for GFP-derived hBMECs cultured with retinoic acid (N = 3) **(Fig 4D)**. The decrease in permeability with the addition of retinoic acid was statistically significant (p < 0.05). The LY permeabilities for hBMECs and hBMECs cultured with retinoic acid are almost identical. The permeability of LY across MDCK and CaCo-2 cells, widely used to assess drug permeability, is typically ≤ 1 x 10^−6^ cm s^-1^ in PTFE inserts using HBSS [39]. LY permeabilities of 1 x 10^−6^ to 1 x 10^−5^ cm s^-1^ have been reported for immortalized HBMECs using PET inserts in Ringer HEPES buffer [11]. The LY permeabilities for the hBMECs are amongst the lowest reported in the literature, highlighting the potential of these cells for drug discovery.

The permeability of tacrine was 9.39 ± 0.54 x 10^−6^ cm s^-1^ for hBMECs, 7.09 ± 2.78 x 10^−6^ cm s^-1^ for hBMECs with the addition of retinoic acid, and 7.66 ± 1.02 x 10^−6^ cm s^-1^ for GFP-labeled hBMECs treated with retinoic acid (N = 3) (**Fig 4D)**. The addition of retinoic acid to the hBMECs did not significantly influence tacrine permeability, nor did the use of the BC1-GFP-derived cells (p > 0.05). The reported permeability for tacrine across MDCK cells was 6 x 10^−5^ cm s^-1^ (polycarbonate 12 well inserts using HBSS) [8], 6- to 9-fold higher than for the hBMECs.

The apical-to-basolateral permeability of rhodamine 123 was 7.32 ± 2.65 x 10^−6^ cm s^-1^ for hBMECs, 5.69 ± 2.73 x 10^−7^ cm s^-1^ for hBMECs with the addition of retinoic acid, and 3.23 ± 0.40 x 10^−7^ cm s^-1^ for GFP-labeled hBMECs cultured with retinoic acid (N = 3) (**Fig 4D)**. The basolateral-to-apical permeability was 1.59 ± 0.74 x 10^−5^ cm s^-1^ for hBMECs, 2.06 ± 0.68 x 10^−6^ cm s^-1^ for hBMECs cultured with retinoic acid, and 6.03 ± 0.79 x 10^−7^ cm s^-1^ for GFP-labeled hBMECs treated with retinoic acid (N = 3) (**Fig 4D)**. The addition of retinoic acid did not significantly change the apical-to-basolateral or basolateral-to-apical permeability of rhodamine 123. The reported apical-to-basolateral and basolateral-to-apical permeabilities of rhodamine 123 across MDCKII.MDR1 monolayers transfected to overexpress P-gp were 0.8 x 10^−6^ cm s^-1^ and 7.6 x 10^−6^ cm s^-1^, respectively (polycarbonate 12 well inserts in HBSS) [43].

The calculated efflux ratios for rhodamine 123 across monolayers of the derived cells were 2.10 ± 0.64 for hBMECs, 3.96 ± 0.30 for hBMECs cultured with retinoic acid, and 1.82 ± 0.57 for GFP-labeled hBMECs cultured with retinoic acid, significantly higher (p < 0.05) than the efflux ratio of 0.984 ± 0.19 for LY across hBMECs cultured with retinoic acid. There was no difference between cells cultured with retinoic acid, or those transfected with GFP. These efflux values are similar the value of 9 reported for MDCKII.MDR1 cells transfected to overexpress P-gp [43]. The efflux ratios indicate polarization of efflux transporters, which is a known characteristic of brain capillaries [44].

### Functionality: capillary organization

**Fig 5:**
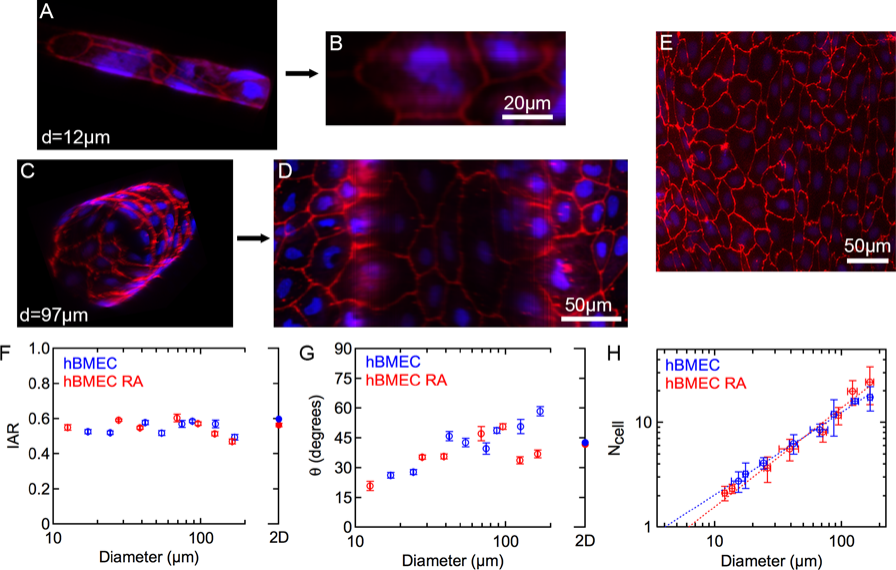
Influence of curvature on morphology of hBMECs. DAPI (blue), ZO-1 (red). (A) Confocal microscopy image of hBMECs seeded on a 12 μm diameter glass rod. (B) Corresponding unwrapped 2D projection. (C) Confocal microscopy image of hBMECs seeded on a 97 μm diameter glass rod. (D) Corresponding unwrapped 2D projection. (E) Fluorescence image of a 2D monolayer of hBMECs. (F) Inverse aspect ratio of hBMECs on rods of varying diameters. (G) Average orientation angle of hBMECs versus rod diameter. (H) Number of cells wrapping around the perimeter versus rod diameter. The dotted lines show a linear least squares fit to a power law (N_cell_ ∝ d^α^) where α = 0.79 for hBMECs and α = 0.94 for hBMECs with retinoic acid. Error bars for both axes represent SE. Data was obtained from analysis of 1756 hBMEC cells and 2149 hBMEC cells cultured with retinoic acid.

In blood vessels, the 3D cylindrical geometry is characterized by the curvature, which is defined by the inverse of the vessel radius (1/r, where r is the vessel radius). In the human brain, hBMECs form capillaries with a diameter of 8–10 μm by wrapping around to form tight junctions with themselves and their upstream and downstream neighbors [2]. To assess the response of hBMECs to the high curvature associated with small vessels and capillaries we used the rod assay [23]. In this assay, cells are grown to confluence on rods with diameters from 10 μm to 200 μm spanning the range from brain capillaries to larger vessels. The derived hBMECs are stained for ZO-1 to visualize the cell-cell junctions (**Fig 5A and 5C**), and confocal microscope images are transposed to produce 2D projections (**Fig 5B and 5D**) for quantitative morphological analysis [23]. Morphological parameters, including the inverse aspect ratio (IAR), the average orientation angle, and the number of cells around the perimeter (**Fig 5**), are then determined from analysis of confocal microscope images. The IAR is a measure of the elongation of the cells, defined as the length of the short axis divided by the length of the long axis. The average orientation angle is a measure of the axial alignment of the cells and is defined by the angle (0–90˚) between the long axis of the cell and the rod axis. Data for 2D monolayers are provided for comparison. For the smallest diameter rods, about 10 μm, the hBMECs were found to wrap around the rods to form tight junctions with themselves as well as their neighbors (**Fig 5B**), similar to the morphology in brain capillaries [2].

The inverse aspect ratio for hBMECs in confluent monolayers with and without retinoic acid was generally in the range 0.5–0.6, with no dependence on diameter (**Fig 5F**), indicating that BC1-derived hBMECs do not elongate due to curvature. The average orientation angle for hBMECs in confluent monolayers in 2D was around 45˚, as expected for a random distribution between 0˚ and 90˚ with no preferred alignment (**Fig 5G**). The average orientation angle decreased with decreasing rod diameter, reaching a value of around 20˚ at very small diameters (**Fig 5G**). The response of hBMECs cultured with and without retinoic acid was indistinguishable. This decrease shows that the hBMECs undergo some alignment with decreasing diameter, with the long axis of the cells becoming more oriented with the rod axis.

To investigate how hBMECs are organized in microvessels as the diameter decreases, the average number of cells around the perimeter of the rods was analyzed (**Fig 5H**). The number of cells around the perimeter decreases with decreasing diameter following a power law (N_cell_ ∝ d^α^) with an exponent α = 0.79 for hBMECs without retinoic acid and α = 0.94 for hBMECs treated with retinoic acid (**Fig 5H**). For a fixed cell shape (e.g. IAR) and area, the exponent α is expected to be 1.0, and the exponents measured for hBMECs are close to the expected value. Extrapolation of the curves to N_cell_ = 1 provides an estimate for the diameter at which all cells are expected to wrap around and form tight junctions with themselves. From the linear least squares fits, we obtain critical diameters of 4 μm for hBMECs and 6 μm for hBMECs cultured with retinoic acid, very close to the diameter of capillaries in the human brain of around 8 μm [2].

These results show that BC1-derived hBMECs cultured with and without retinoic acid do not elongate but show alignment in response to the high curvature associated with capillary diameters. The alignment, as evidenced by the decrease in orientation angle with decreasing rod diameter, is not a result solely due to a finite size effect (**S2 Fig**). In previous work, we have shown that immortalized hBMECs do not elongate or align even at small diameters, whereas human umbilical vein endothelial cells (HUVECs), representative of larger vessels, become progressively elongated and aligned with decreasing diameter, with an IAR < 0.3 and an orientation angle of < 5˚ on 10 μm diameter rods [23]. Therefore, the stem-cell derived hBMECs exhibit a slightly different response to curvature compared to the immortalized hBMECs. Taken together, these results suggest that the ability to wrap around in regions of high curvature (small vessels) is a phenotype of brain microvascular endothelial cells.

## Discussion

We have derived hBMECs from the BC1 and BC1-GFP human iPS cell lines using the approach developed by Lippmann et al. [14, 17]. The differentiation protocol is highly reproducible and can be performed with a consistently high yield as long as: (1) the differentiation (step 1) is initiated at the optimum time, and (2) the maturation of the endothelial cells during differentiation (step 1) is terminated at the optimum time. Non-optimum timing resulted in variations in differentiation and poorer phenotypes. With optimal timing, the differentiation yields 98.3 ± 1.2% PECAM^+^ endothelial cells with high GLUT-1^+^ expression, indicating a high purity of brain phenotypic endothelial cells (PECAM-1^+^/GLUT-1^+^).

We characterized protein and gene expression, barrier function, and the capillary organization for hBMECs derived from both iPSC lines. We also examined the influence of retinoic acid on the derived cells. It has been suggested that retinoic acid induces the blood-brain barrier endothelial phenotype during development [45] and Lippmann et al. have reported improvements in the differentiation of hBMECs with addition of retinoic acid [17]. The hBMECs derived from the BC1 and BC1-GFP cell lines recapitulate many of the features reported by Lippmann et al. that are characteristic of the blood-brain barrier phenotype, including expression of tight junction proteins, efflux pumps, and transporters, high transendothelial electrical resistance (TEER), low permeability, and polarized P-gp efflux pumps.

From immunofluorescence images, the tight junction proteins claudin-5, occludin, and ZO-1 are observed at the cell-cell junctions for hBMECs derived from both iPS cell lines with and without retinoic acid (**Fig 2**). The junctions form a network of segments that are relatively straight with few frayed strands. There was no significant difference in expression of claudin-5, the P-gp efflux pump, or the LAT-1 transporter for hBMECs derived from either iPS cell line with and without retinoic acid (**Fig 3B**). In contrast, the expression of the adherens junction protein, VE-cadherin, was significantly upregulated in both the BC1 and BC1-GFP cell lines with the addition of retinoic acid (**Fig 3B**). This is in agreement with Lippmann et al. who reported a similar increase in expression of VE-cadherin with the addition of retinoic acid [17], but also observed a decrease in claudin-5 expression and an increase in occludin expression that was not observed here. Flow cytometry results also confirm expression of endothelial markers, von Willebrand factor (vWF), and PECAM-1, as well as tight junction markers, occludin and claudin-5.

The expression of genes encoding for junctional proteins, claudin-5, ZO-1, VE-cad, and occludin, and transporters, P-gp, BCRP, MRP1, GLUT-1 and LAT-1, showed no significant differences between the BC1 and BC1-GFP cell lines, or between cell lines cultured with retinoic acid (**Fig 3C and 3D**). While the average expression of the P-gp gene was several fold higher for both cell lines with the addition of retinoic acid, the variability between different differentiations was also high, resulting in no statistical significance. Despite the large variability in expression of the *CLDN5*, *ABCB1*, *ABCG2*, and *ABCC1* genes between differentiations, there was no observable difference in the fluorescence intensity or distribution of the corresponding proteins in immunofluorescence images. In addition, the variability in protein expression of the corresponding protein was also much smaller between differentiations.

Overall, results from both protein and gene expression clearly show the expression of tight junction proteins, and in particular claudin-5, along with the P-gp, BCRP, and MRP1 transporters, and the GLUT-1 and LAT-1 transporters, but with no significant difference in expression levels between the BC1 and BC1-GFP cell lines, with or without the addition of retinoic acid.

The addition of retinoic acid significantly increased the TEER values for hBMECs derived from both iPS cell lines, resulting in physiological values close to 2000 Ω cm^2^ (**Fig 4**). As described above, retinoic acid is implicated in the induction of the blood-brain barrier endothelial phenotype during development [45] and Lippmann et al. have also reported physiological TEER values for iPSC-derived hBMECs treated with retinoic acid [17]. The increase in TEER values for cells treated with retinoic acid suggests an increase in the quality of the tight junctions and an associated decrease in paracellular transport, although we do not observe any differences in expression of tight junction proteins. This could be explained by an increase in localization of the tight junction proteins at the cell-cell junctions, although this is not supported by the immunofluorescence images (**Fig 2**). In contrast to cell lines commonly used in blood-brain barrier research and other endothelial cell lines, the iPSC-derived hBMECs exhibit high TEER values and consistent expression of important blood-brain barrier markers (**S2 Table)**.

The permeability of Lucifer yellow for hBMECs derived from both iPS cell lines and cultured in retinoic acid was around 4 x 10^−7^ cm s^-1^ (**Fig 4D**), lower than values reported for MDCK cells or other brain microvascular endothelial cells [8]. The permeability of tacrine was about 1 x 10^−5^ cm s^-1^, similar to the values reported for MDCK cells [8]. The permeability of rhodamine 123 was around 1 x 10^−6^ cm s^-1^ with efflux ratios around 3.

For LY, the permeability is significantly lower for cells treated with retinoic acid. LY crosses the cell membrane by passive transport and hence the decrease in permeability with retinoic acid is unlikely to be related to the decrease in paracellular transport implied by the increased TEER values. The decrease in permeability could be due to changes in cell membrane composition. Evidence suggests that tacrine transport is dominated by organic cationic transporters [46], therefore the similarity in permeability values suggests that retinoic acid does not modulate this transport mechanism. The permeability of LY in the hBMECs cultured in retinoic acid is lower than typically reported values for MDCK cells, and the permeability of tacrine is 6- to 9-fold lower than values reported for MDCK cells. The permeability of rhodamine is similar to values reported for MDCK cells and the efflux ratio of 2–4 indicates polarization of efflux pumps, similar to MDCKs. An important question to be resolved is the whether the lower permeability is representative of the human BBB.

Using the rod assay to assess capillary organization, we show that derived hBMECs resist elongation with decreasing rod diameter (increasing curvature), but show progressive axial alignment (**Fig 5**). In contrast, endothelial cells derived from larger vessels (HUVECs) elongate and align on rods with decreasing diameter. At rod diameters similar to human brain capillaries, single derived hBMECs wrap around to form tight junctions with themselves and their upstream and downstream neighbors, similar to the morphology in brain capillaries [2]. In contrast, HUVECs elongate and align along the rod axis to avoid the high curvature, and hence single cells cannot form junctions with themselves. These results suggest that the ability to resist elongation at high curvature (small diameters) is a phenotype of brain microvascular endothelial cells.

The BC1 and BC1-GFP iPS cell lines use human feeder cells thereby avoiding undesired immunogenicity. Differentiation under xeno-free conditions has the potential for clinical applications [20]. GFP expression is maintained throughout the differentiation of BC1-GFP cells and remains strong in the derived hBMECs. Not all transfected stem cells maintain their GFP expression following differentiation [47]. The expression of GFP in hBMECs derived from the BC1-GFP cell line provides an important new resource for BBB research, allowing easy identification and tracking of these cells both for *in vitro* and *in vivo* applications using fluorescence microscopy.

## Supporting Information

S1 Table Primary antibodies used for staining for immunofluorescence (IF), flow cytometry (FC) and western blots (WB). (DOCX)

S2 Table Comparison of endothelial cell lines commonly used in blood-brain barrier models. (DOCX)

S1 Fig Representative western blots for CLDN-5, P-gp, LAT-1, OCLN, VE-cad, and SOX-2. (DOCX)

S2 Fig Cell length and orientation angles for hBMECs cultured with retinoic acid in confluent monolayers on rods of different diameter. (DOCX)

S3 Fig Cell area for hBMECs and hBMECs cultured with retinoic acid in confluent monolayers on rods with different diameter and in 2D. (DOCX)
