# Supplementary material for: Human Brain Microvascular Endothelial Cells Derived from the BC1 iPS Cell Line Exhibit a Blood-Brain Barrier Phenotype
Source: PLoS One. 2016 Apr 12;11(4):e0152105. doi: 10.1371/journal.pone.0152105 (PMC4829259; doi:10.1371/journal.pone.0152105)
Supplement: S1 Table — (DOCX) [file pone.0152105.s001.docx]

**Supporting Information**

| Target Antigen | Antibody Description | Vendor | Product Number | Dilution |
| --- | --- | --- | --- | --- |
| ZO-1 | rabbit polyclonal | Life Technologies | 40-2200 | 1:100 IF |
| OCLN | mouse monoclonal | Life Technologies | 33-1500 | 1:10 IF  1:10 FC |
| CLDN-5 | mouse monoclonal | Life Technologies | 35-2500 | 1:100 IF  1:10 FC  1:1500 WB |
| PECAM-1 | rabbit polyclonal | Thermo Scientific | RB-10333-P | 1:10 , FC |
| vWF | goat polyclonal | Santa Cruz | sc-8068 | 1:10 FC |
| GLUT1-PE | mouse monoclonal | R&D Systems | FAB1418P |  |
| LAT-1 | rabbit polyclonal | Santa Cruz | sc-134994 | 1:1500 WB |
| P-gp | mouse monoclonal | Sigma-Aldrich | P7965-.2ML | 1:10 FC  1:1500 WB |
| β-actin | rabbit polyclonal | Cell Signaling Technologies | 4967S | 1:3000 WB |
| Goat Anti-Mouse IgG HRP |  | Bio-Rad | 1706516 | 1:1500 WB |
| Goat Anti-Rabbit HRP |  | Bio-Rad | 1721019 | 1:1500 WB |

**Table S1.** Primary antibodies used for staining for immunofluorescence (IF), flow cytometry (FC) and western blots (WB).
