# Supplementary material for: Human Brain Microvascular Endothelial Cells Derived from the BC1 iPS Cell Line Exhibit a Blood-Brain Barrier Phenotype
Source: PLoS One. 2016 Apr 12;11(4):e0152105. doi: 10.1371/journal.pone.0152105 (PMC4829259; doi:10.1371/journal.pone.0152105)
Supplement: S2 Table — (DOCX) [file pone.0152105.s002.docx]

| Cell Type | hBMEC | hBMEC RA | hBMEC | hBMEC RA | HUVEC | MDCK.II | D3 | H2 | BAE | BLEC |
| --- | --- | --- | --- | --- | --- | --- | --- | --- | --- | --- |
| Properties |  |  |  |  |  |  |  |  |  |  |
| Species | Human | Human | Human | Human | Human | Canine | Human | Human | Bovine | Human |
| Origin | iPSC derived | iPSC derived | iPSC derived | iPSC derived | Umbilical cord | Imm. kidney | Imm. brain | Imm. brain | Aorta | HSC derived |
| Functionality |  |  |  |  |  |  |  |  |  |  |
| TEER  (Ω cm^2^) | 200 | 2000 | 230 | 2000 | 74 | 200 | 10-20 | 35 | 13-40 | 180 |
| Markers |  |  |  |  |  |  |  |  |  |  |
| Claudin-5 | + | + | + | + | + | - | - | + | - | + |
| ZO-1 | + | + | + | + | + | + | + | + | + | + |
| Occludin | + | + | + | + | - | + | +/- | + | + | + |
| P-gp | + | + | + | + |  | T | + |  |  | + |
| VE-cad | +/- | + | +/- | + | + | - | + | + | + | + |
| References | this  work | this  work | [1] | [1, 2] | [3-6] | [7-9] | [10, 11] | [10] | [12-14] | [15] |

**Supporting Information**

**Table S2.**  Comparison of endothelial cell lines commonly used in blood-brain barrier models. hBMECs derived from iPSCs have high TEER, expression of tight junction proteins, efflux transporters, and nutrient transporters. Other endothelial cell lines show low TEER and do not express all tight junction proteins. hBMEC – human brain microvascular endothelial cell derived from iPSCs, RA – retinoic acid, HUVEC – human umbilical vein endothelial cells, MDCK - Madin-Darby canine kidney cells, BAE – bovine aortic endothelial cells, BLEC - brain-like endothelial cells derived from cord blood-derived hematopoietic stem cells. Imm. – immortalized. T – transfected.

**References**

[1] Lippmann ES, Azarin SM, Kay JE, Nessler RA, Wilson HK, Al-Ahmad A, et al. Derivation of blood-brain barrier endothelial cells from human pluripotent stem cells. Nat Biotechnol. 2012;30:783-91.

[2] Lippmann ES, Al-Ahmad A, Azarin SM, Palecek SP, Shusta EV. A retinoic acid-enhanced, multicellular human blood-brain barrier model derived from stem cell sources. Sci Rep. 2014;4:4160.

[3] Mann GE, Yudilevich DL, Sobrevia L. Regulation of amino acid and glucose transporters in endothelial and smooth muscle cells. Physiological reviews. 2003;83:183-252.

[4] Müller AM, Hermanns MI, Skrzynski C, Nesslinger M, Müller K-M, Kirkpatrick CJ. Expression of the endothelial markers PECAM-1, vWf, and CD34 in vivo and in vitro. Experimental and molecular pathology. 2002;72:221-9.

[5] Man S, Ubogu EE, Williams KA, Tucky B, Callahan MK, Ransohoff RM. Human brain microvascular endothelial cells and umbilical vein endothelial cells differentially facilitate leukocyte recruitment and utilize chemokines for T cell migration. Clinical and Developmental Immunology. 2008;2008.

[6] Clark PR, Kim RK, Pober JS, Kluger MS. Tumor Necrosis Factor Disrupts Claudin-5 Endothelial Tight Junction Barriers in Two Distinct NF-κB-Dependent Phases. PloS one. 2015;10.

[7] Piehl C, Piontek J, Cording J, Wolburg H, Blasig IE. Participation of the second extracellular loop of claudin-5 in paracellular tightening against ions, small and large molecules. Cellular and Molecular Life Sciences. 2010;67:2131-40.

[8] Wilhelm I, Fazakas C, Krizbai IA. In vitro models of the blood-brain barrier. Acta Neurobiol Exp (Wars). 2011;71:113-28.

[9] Stevenson BR, Anderson JM, Goodenough DA, Mooseker MS. Tight junction structure and ZO-1 content are identical in two strains of Madin-Darby canine kidney cells which differ in transepithelial resistance. The Journal of cell biology. 1988;107:2401-8.

[10] Eigenmann DE, Xue G, Kim KS, Moses AV, Hamburger M, Oufir M. Comparative study of four immortalized human brain capillary endothelial cell lines, hCMEC/D3, hBMEC, TY10, and BB19, and optimization of culture conditions, for an in vitro blood-brain barrier model for drug permeability studies. Fluids Barriers CNS. 2013;10:33.

[11] Weksler B, Romero IA, Couraud PO. The hCMEC/D3 cell line as a model of the human blood brain barrier. Fluids Barriers CNS. 2013;10:16.

[12] Pekala P, Marlow M, Heuvelman D, Connolly D. Regulation of hexose transport in aortic endothelial cells by vascular permeability factor and tumor necrosis factor-alpha, but not by insulin. Journal of Biological Chemistry. 1990;265:18051-4.

[13] Rubin L, Staddon J. The cell biology of the blood-brain barrier. Annual review of neuroscience. 1999;22:11-28.

[14] Rutten MJ, Hoover RL, Karnovsky MJ. Electrical resistance and macromolecular permeability of brain endothelial monolayer cultures. Brain research. 1987;425:301-10.

[15] Cecchelli R, Aday S, Sevin E, Almeida C, Culot M, Dehouck L, et al. A Stable and Reproducible Human Blood-Brain Barrier Model Derived from Hematopoietic Stem Cells. Plos One. 2014;9.
