# Supplementary material for: Human Brain Microvascular Endothelial Cells Derived from the BC1 iPS Cell Line Exhibit a Blood-Brain Barrier Phenotype
Source: PLoS One. 2016 Apr 12;11(4):e0152105. doi: 10.1371/journal.pone.0152105 (PMC4829259; doi:10.1371/journal.pone.0152105)
Supplement: S1 Fig — (DOCX) [file pone.0152105.s003.docx]

**Supporting Information**

**
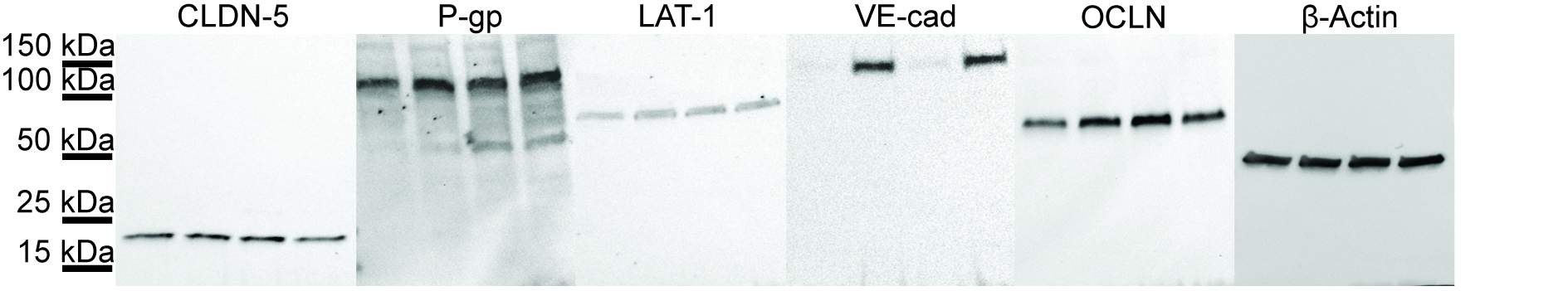
**

**
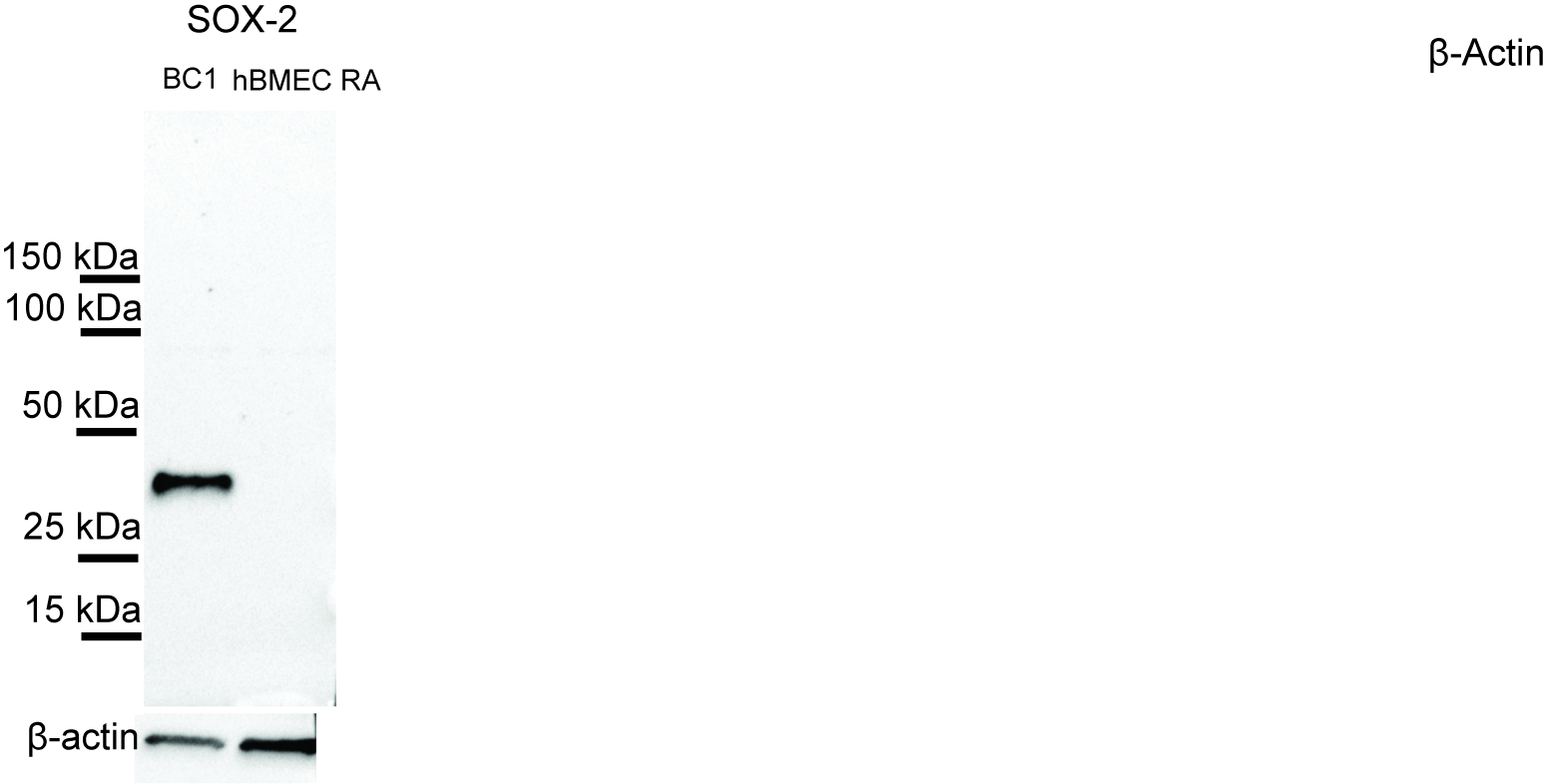
**

**Figure S1.** Representative western blots for CLDN-5, P-gp, LAT-1, OCLN, VE-cad, and SOX-2.

(**Top**) Western blots for claudin-5, p-glycoprotein, large amino acid transporter 1, vascular endothelial cadherin, occludin, and β-actin on (lanes left to right): hBMEC, hBMEC RA, hBMEC GFP, and hBMEC GFP RA. CLDN-5, P-gp, LAT-1, and OCLN have similar expression in all four cell types. VE-cad expression is significantly increased in cells treated with retinoic acid (both hBMEC RA and hBMEC GFP RA).

**(Bottom)** Western blots for SOX-2 before and after the differentiation. SOX-2 is a commonly used marker of stem and is expressed in neural precursor cells, loss of SOX-2 expression is associated with the loss of potential to differentiate further [1]. BC1-derived hBMECs do not show a visible band indicating the expression of SOX-2, demonstrating the loss of stem markers during the differentiation.

**References**

[1] Hutton SR, Pevny LH. SOX2 expression levels distinguish between neural progenitor populations of the developing dorsal telencephalon. Developmental biology. 2011;352:40-7.
