# Supplementary material for: Human Brain Microvascular Endothelial Cells Derived from the BC1 iPS Cell Line Exhibit a Blood-Brain Barrier Phenotype
Source: PLoS One. 2016 Apr 12;11(4):e0152105. doi: 10.1371/journal.pone.0152105 (PMC4829259; doi:10.1371/journal.pone.0152105)
Supplement: S2 Fig — (DOCX) [file pone.0152105.s004.docx]

**Supporting Information**


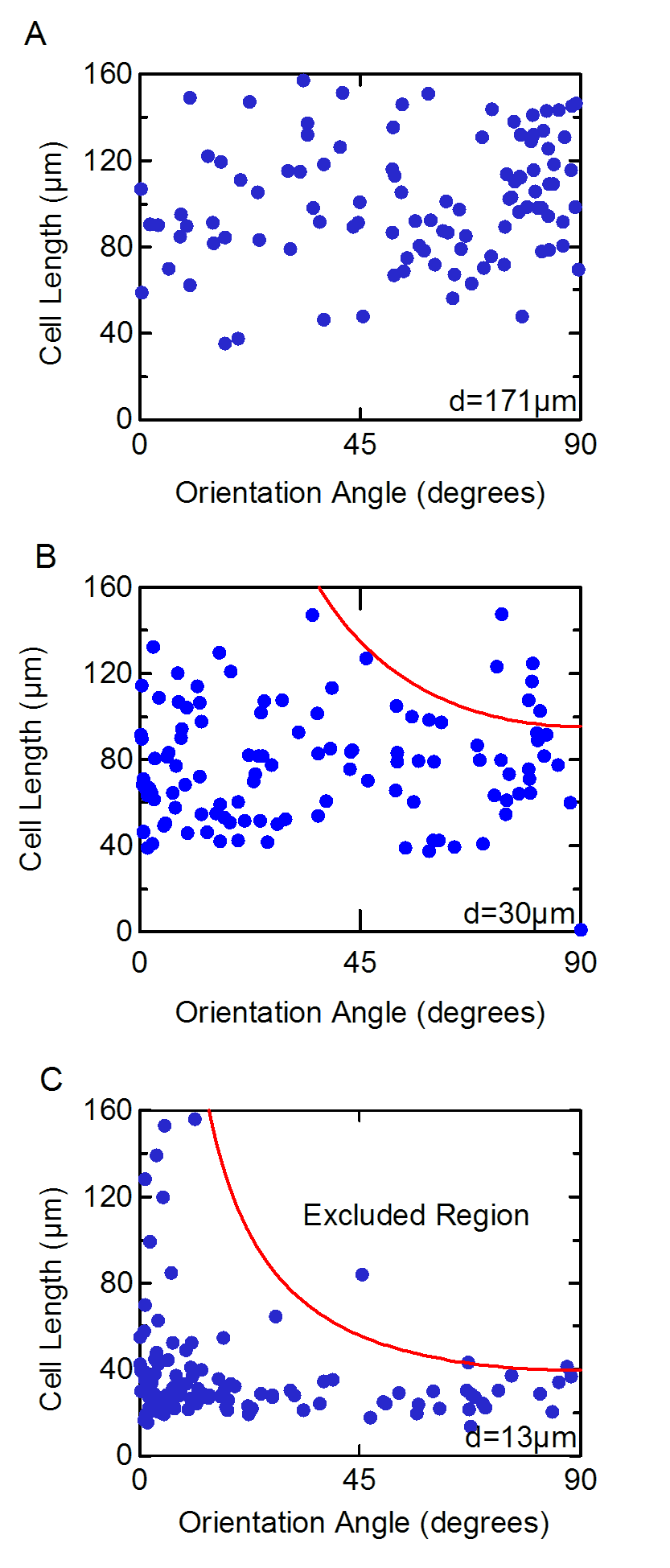


**Figure S3**. Cell length and orientation angles for hBMEC RA cells in confluent monolayers on rods of different diameter. (A) Large diameter rods (171.4 ± 0.22 µm (SE), N = 119), (B) small diameter rods (30.4 ± 0.02 µm (SE), N= 115), and (C) very small diameter rods (12.6 ± 0.08 µm (SE), N = 118). The solid red lines represents $l_{cell}\sin\theta= \pi\text{d}$.

On large diameter rods (A), cells can adopt any orientation angle and exhibit the full range of allowed angles and cell lengths. On smaller diameter rods (B,C), cells are limited to angles where $l_{cell}\sin\theta\leq\pi\text{d}$ (solid red line) due to finite size effects. On very small diameter rods (C), the cell length does not approach the excluded region due to cell alignment along the rod axis.

The orientation angle is defined as the angle between the major axis of the cell and the rod axis. In the case of larger diameter rods, where the perimeter (πd) is much greater than the length of the cell, the cells can orient themselves in any angle between 0° and 90°. Assuming an even distribution, the average orientation angle for large diameter rods should be 45° if there is no preferential alignment of the cells to the rod axis. However, in the case of small diameter rods where the length of the cell is greater than the perimeter (πd), the cells cannot orient themselves in the full range of angles—large angles are not possible. Therefore, given an uniform distribution, the average orientation angle for small diameter rods is less than 45°.

Scatter plots of cell length and orientation angle for individual cells on large diameter rods (171.4 ± 0.22 µm (SE)) and on a small diameter rods (12.6 ± 0.08 µm (SE)) are plotted. On the large diameter rods, the cells have a random orientation resulting in a uniform distribution (Figure S2A). On the small diameter rods, the range of angles for cells with long cell length is restricted (Figure S2B). The solid red line is $l_{cell}\sin\theta= \pi\text{d}$ where $l_{cell}$ is the length of the major axis of the cell, θ is the orientation angle of the cell, and d is the diameter of the rod. Cells can adopt any orientation as long as $l_{cell}\sin\theta\leq\pi\text{d}$.
