# Supplementary material for: Human Brain Microvascular Endothelial Cells Derived from the BC1 iPS Cell Line Exhibit a Blood-Brain Barrier Phenotype
Source: PLoS One. 2016 Apr 12;11(4):e0152105. doi: 10.1371/journal.pone.0152105 (PMC4829259; doi:10.1371/journal.pone.0152105)
Supplement: S3 Fig — (DOCX) [file pone.0152105.s005.docx]

**Supporting Information**


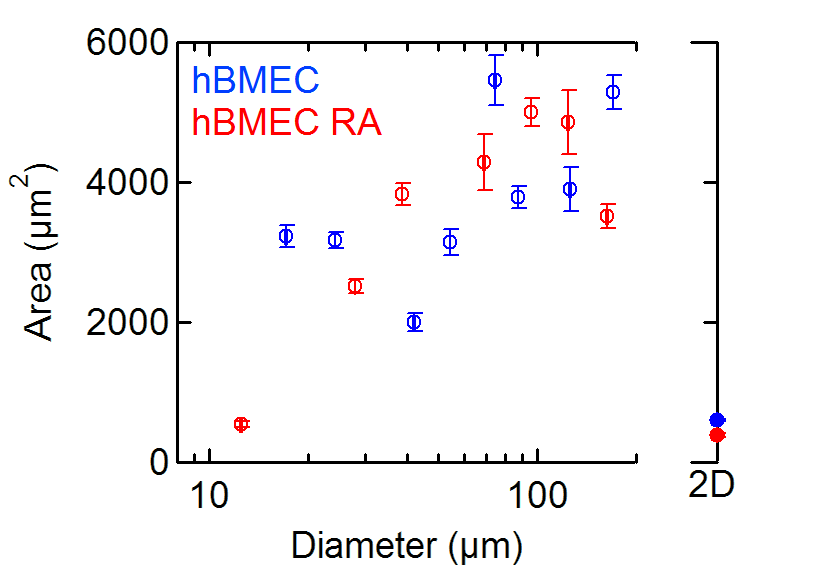


**Figure S3.** Cell area for hBMECs and hBMEC-RA in confluent monolayers on rods with different diameter and in 2D. Least squares fits show no trend between diameter of rod and area (EC: R^2^  = 0.4476, RA: R^2^ = 0.0824). Cells were seeded at 1 x 10^6^ cells mL^-1^. The difference in area between cells in 2D and on large diameter rods is due to the fact that in the 2D experiments the cells were confined to the size of the dish, whereas in the rod experiments excess cells could detach from the suspended glass rods.
